# Supplementary material for: Comparative multiomics analysis of cell physiological state after culture in a basket bioreactor
Source: Sci Rep. 2022 Nov 23;12:20161. doi: 10.1038/s41598-022-24687-4 (PMC9686226; doi:10.1038/s41598-022-24687-4)
Supplement: Supplementary file 1 — Supplementary Information 1. [file 41598_2022_24687_MOESM1_ESM.zip › raw data/Proteomic raw data/6.KEGGenrichment/EG.vs.CG/ALL/pathway/src/00620.html]

map00620


- K00626, 2.3.1.9, R00238"

- Up regulated proteins

A0A0D9S9P1(Description:Uncharacterized protein;Gene:;log2FC:1.9645)

A0A0D9RJH6(Description:Uncharacterized protein;Gene:;log2FC:1.7929)

Close
